# Lactoferrin Alleviates Inflammation and Regulates Gut Microbiota Composition in H5N1-Infected Mice

**DOI:** 10.3390/nu15153362

**Published:** 2023-07-28

**Authors:** Yanyi Huang, Peiyang Zhang, Shuyi Han, Hongxuan He

**Affiliations:** 1 National Research Center for Wildlife-Borne Diseases, Institute of Zoology, Chinese Academy of Sciences, Beijing 100101, China; 2 College of Life Science, University of Chinese Academy of Sciences, Beijing 100101, China

**Keywords:** lactoferrin, H5N1, influenza, inflammation, gut microbiota

## Abstract

The impact of lactoferrin, an antimicrobial peptide (AMP) with iron-binding properties, on the intestinal barrier and microflora of mice infected with highly pathogenic avian influenza A (H5N1) virus remains unclear. To investigate the effects of lactoferrin on the histopathology and intestinal microecological environment, we conducted a study using H5N1-infected mice. H5N1 infection resulted in pulmonary and intestinal damage, as well as an imbalance in gut microbiota, significantly increasing the abundance of pathogenic bacteria such as *Helicobacter pylori* and *Campylobacter*. The consumption of lactoferrin in the diet alleviated lung injury and restored the downregulation of the INAVA gene and intestinal dysfunction caused by H5N1 infection. Lactoferrin not only reduced lung and intestinal injury, but also alleviated inflammation and reversed the changes in intestinal microflora composition while increasing the abundance of beneficial bacteria. Moreover, lactoferrin rebalanced the gut microbiota and partially restored intestinal homeostasis. This study demonstrated that lactoferrin exerts its effects on the intestinal tract, leading to improvements in gut microbiota and restoration of the integrity of both the intestinal wall and lung tissue. These findings support the notion that lactoferrin may be a promising candidate for systemic treatment of influenza by locally acting on the intestine and microbiota.

## 1. Introduction

Antimicrobial peptides (AMPs) are a diverse group of bioactive small proteins with multifaceted activities, which include the ability to favorably modulate the host immune response [1,2]. Over the past few decades, AMPs have emerged as a powerful class of alternative drugs, meeting the need for novel anti-infective agents to address the growing problem of antibiotic resistance [3,4]. Lactoferrin is an important multifunctional glycoprotein, which exists in the milk of mammals and other secretions. Lactoferrin has a broad spectrum of antibacterial, anti-inflammatory, anti-tumor and immunomodulatory functions [5]. Under acidic conditions, such as the degradation of pepsin, lactoferrin produces small molecular antibacterial peptides [6], which can exert antibacterial and anti-inflammatory effects in a variety of ways. In addition, it also promotes the growth of intestinal villi [7]. Studies have confirmed that lactoferrin addition to feed increased intestinal function and promoted the repair of intestinal injury [8].

The mammalian gut microbiome is a dynamic microbiome, consisting of a diverse array of viruses, bacteria and fungi [9]. These microbiomes are increasingly found to be key regulators of body health and diseases [10,11]. In addition, the microbiome provides nutrients necessary for the host body to maintain normal life activities through its own metabolism [12]. They can also form a relatively stable intestinal microecology, which also behaves as a bacterial membrane barrier that resists the colonization of foreign microorganisms, and maintains the original steady-state environment. When normal microbial homeostasis is disrupted, it is easy to develop a series of serious metabolic disorders and diseases, such as cardiovascular disease and inflammatory bowel disease [13,14,15], suggesting that intestinal microbiome disorders lead to impaired immune function of the host body. Moreover, studies have shown that these microorganisms also act as important regulators of intestinal mucosal barrier repair [16,17], further supporting anti-pathogens defense in the host body [18].

Influenza A virus infection in the respiratory tract first affects the lung epithelial cells, leading to inflammation in a large number of cells, which flood in the infected area, resulting in severe immune response and may even destroy the normal cell structure and function [19]. The mucosal immune system plays an important role in the immune regulation of the body [20]. In influenza infection, both lung and intestinal damage are caused by the connection between the two mucosal sites [21]. It has been previously reported in a mouse model of respiratory influenza infection that the influenza virus was not detected in the intestinal tract, and no intestinal immune damage was caused by injecting the influenza virus directly into the intestine [22]. After influenza infection, the percentage and number of CCR9^+^CD4^+^ T cells increase in the lungs, which in turn specifically migrate to the intestinal mucosa through CCL25-CCR9 chemokine axis [23], producing interferon (IFN)-γ and causing intestinal damage. Additionally, an increase in the number of neutrophils is thought to be the cause of acute lung injury caused by influenza, and interleukin (IL)-17 regulates neutrophil recruitment. Influenza infection can change the composition of intestinal flora, promote the polarization of T helper 17 (Th17) cells in the small intestine and produce IL-17, thereby causing intestinal immune damage [24]. Although lactoferrin showed an anti-inflammatory effect in inflammatory bowel disease [25], the application of lactoferrin in the treatment of H5N1 infection remains unexplored. Consequently, the present study was conducted to explore the effects of lactoferrin on inflammation in a mouse model of H5N1 infection. Furthermore, whether microbes were involved in and mediated the beneficial effects of lactoferrin were also explored.

## 2. Materials and Methods

### 2.1. Viruses

The H5N1 virus strain A/environment/Qinghai/1/2008 was isolated from a water environment in Qinghai and stored at −80 °C after passaging in 9–10-day embryonated chicken eggs. Virus titer, as a 50% Tissue Culture Infectious Dose (10^8.11^ TCID_50_/mL), was determined in Madin-Darby Canine Kidney (MDCK) cells.

### 2.2. Antimicrobial Peptide Diets

The maintenance feed for mice was purchased from the SPF (Beijing) Biotechnology Co. Ltd. Lactoferrin feed additive was obtained from the Guangzhou Glam Biotechnology Co. Ltd., and was sourced from bovine origin. An amount of 10 mg/day lactoferrin was added to the diet for one mouse.

### 2.3. Animal Experiments

First, 7–8-week-old male BALB/c mice were purchased from SPF Biotechnology Co. Ltd. (Beijing, China), with an average body weight of 25.0 ± 1.0 g. All mice were raised under a 12Light:12Dark (lights on at 9:00 am, off at 9:00 pm) photoperiod and fed with maintenance feed obtained from SPF Biotechnology Co. Ltd before the experiment started. A total of 20 mice were randomly divided into 4 groups with 5 mice per group housing, respectively, in 4 cages. Mice in group 1 received sterile PBS as the control and were only fed with maintenance feed until the end of the experiment (designed as H5N1^−^Lac^−^). Mice in group 2 received sterile PBS and were switched to feed with the AMP diet containing lactoferrin (designed as H5N1^−^Lac^+^). Mice in group 3 were anesthetized via isoflurane inhalation and intranasally infected with 50 µL of the H5N1 virus on day 0; they were continuously fed the maintenance feed without lactoferrin (designed as H5N1^+^Lac^−^). Mice in group 4 were infected in the same way as the mice in group 3, and the feed for this group was replaced with the AMP diet containing lactoferrin until the end of the experiment (designed as H5N1^+^Lac^+^). Body weight and clinical signs were monitored every day. On day 8, all mice were killed to harvest their lungs and colons. The colon surface was sterilized, and contents were harvested into a sterile 2 mL-tube and stored at −80 °C. All animal experiments were performed in animal biosafety level 3 (ABSL3) conditions. All mice received humane care in compliance with the “Guide for the Care and Use of Laboratory Animals” published by the National Institutes of Health. The animal study protocol was approved by the Committee on the Ethics of Animal Experiments of the Institute of Zoology, Chinese Academy of Sciences (approval number: IOZ-IACUC-2022-251).

### 2.4. Histopathology

Lungs and colons (about 1 cm length of distal colon) were fixed with 10% neutral buffered formalin, and were proceeded for paraffin embedding. Paraffin blocks for light microscopy were sectioned as 4 μm thick using a Leica RM2235 microtome (Wetzlar, Germany). Sections were counter-stained with hematoxylin and eosin (H&E). Images were captured using a microscope (Nikon Ci-S, Tokyo, Japan) and viewed using an imaging system (Nikon DS-U3, Tokyo, Japan).

### 2.5. 16S rRNA Library Construction

Total genome DNA from 300 mg of colon content was extracted using the CTAB method. DNA concentration was measured by Nanodrop (Thermo Scientific, Waltham, MA, USA) and DNA molecular size was estimated by 1% agarose gel electrophoresis. The diluted DNA concentration was 1 ng/µL. The 16S rRNA gene region was amplified with a pair of universal primers (forward primer 338F: ACTCCTACGGGAGGCAGCAG and reverse primer 806R: GGACTACHVGGGTWTCTAAT). PCR reaction mixtures contained 10 ng DNA template, 0.2 μM of each primer and 15 µL of Phusion High-Fidelity PCR Master Mix (New England Biolabs, Ipswich, MA, USA). The amplification was carried out in a thermal cycler. The cycling conditions were 98 °C for 1 min, 30 cycles of 98 °C for 10 s, 50 °C for 30 s, 72 °C for 30 s with a final extension step of 72 °C for 5 min. The PCR products were purified using a Qiagen Gel Extraction Kit (Hilden, Germany).

### 2.6. Library Sequencing and Data Analysis

Sequencing libraries were prepared with a NEBNext^®^ Ultra™ IIDNA Library Prep Kit (New England Biolabs) according to the manufacturer’s instructions. The quality of the library was evaluated using Qubit@ 2.0 Fluorometer (Thermo Scientific) and the Agilent Bioanalyzer 2100 system. Sequencing was carried out on an Illumina NovaSeq platform.

Paired-end reads were truncated by cutting off unique barcodes and primer sequences. Paired-end reads were merged using FLASH (Version 1.2.11, http://ccb.jhu.edu/software/FLASH/, accessed on 19 August 2022). Quality filtering on the raw tags was performed using fastp (Version 0.20.0) software to obtain high-quality clean tags. Clean tags were compared with two reference databases (Silva database, https://www.arbsilva.de/, accessed on 25 August 2022, for 16S/18S; Unite database, https://unite.ut.ee/, accessed on 25 August 2022, for ITS) using Vsearch (Version 2.15.0) to detect the chimera sequences. The chimera sequences were removed to obtain effective tags. For the effective tags that had been previously obtained, denoise was performed with DADA2 or the deblur module in QIIME2 software (Version QIIME2-202006) to obtain initial ASVs (Amplicon Sequence Variants), and then ASVs with an abundance of less than 5 were filtered out. Species annotation was performed using QIIME2 software. To study the phylogenetic relationship of each ASV and the differences between the dominant species among different samples (groups), multiple sequence alignment was performed using QIIME2 software. Subsequent analyses of alpha diversity and beta diversity were all performed based on the output normalized data.

### 2.7. Gene Expression Analysis

Total RNA of the lung and colon was extracted using TRIzol reagent. RNA was reverse transcribed into cDNA using a Promega Reverse Transcription System Kit and stored at −20 °C. Specific gene expression was measured using a SYBR-based real-time quantitative PCR assay in the Applied Biosystems 7500 System. Each reaction contained 1 μL cDNA, 0.5 μL of forward and reverse primers, 8 μL of nuclease-free water and 10 μL of SYBR mixture. The cycling conditions were as follows: 95 °C for 3 min followed by 40 cycles of 95 °C for 10 s and 60 °C for 30 s. The relative mRNA expression levels of target genes were calculated using the 2^−ΔΔCt^ method normalized to the internal control GAPDH. The primers used in this study are listed in Table 1.

### 2.8. Statistical Analysis

GraphPad Prism 8 (Graph Pad Software, Inc., San Diego, CA, USA) was used for statistical analyses. Student’s *t*-test was performed to compare mean values between two groups, and one-way ANOVA was followed by the Sidak post hoc test to compare the mean values between multiple groups. All results are presented as mean ± S.D. and a *p*-value < 0.05 was considered statistically significant.

## 3. Results

### 3.1. Effect of Lactoferrin on the Body Weight Change in Mice Infected with H5N1 Influenza Virus

**Figure 1:**
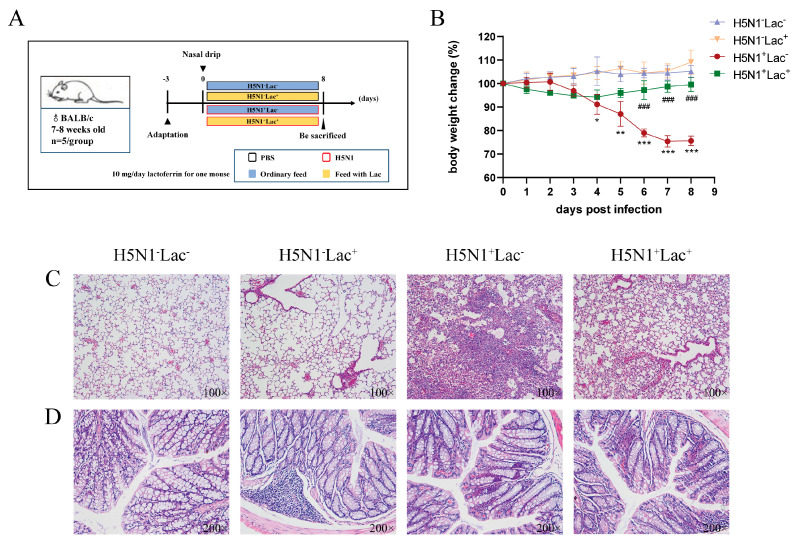
Lactoferrin alleviated the clinical manifestations of the H5N1-infected mice. (**A**) Experimental design. (**B**) Percentage of initial body weight change from the four groups. Values are presented as mean ± S.D. (* *p* < 0.05, ** *p* < 0.01 and *** *p* < 0.001 vs. H5N1^−^Lac^−^ group; ### *p* < 0.001 vs. H5N1^+^Lac^−^ group, *n* = 5 mice per group). (**C**) H&E staining of lung tissue (100×). (**D**) H&E staining of colon tissue (200×).

To evaluate the therapeutic potential of lactoferrin against influenza A viruses, we intranasally inoculated mice with the H5N1 virus and fed them a diet containing lactoferrin (Figure 1A). The results demonstrated that lactoferrin had a significant effect on the body weight recovery of infected mice (Figure 1B). Mice infected with the H5N1 virus lost weight to varying degrees after infection, while uninfected mice had no significant changes in body weight. After being fed with lactoferrin, infected animals began to regain weight and all mice survived before being killed. However, infected mice fed with a standard chow diet without lactoferrin significantly lost weight (the day 8 weight change mean was −24.35% ± 2.07% of baseline weight). Mice inoculated with the H5N1 virus and fed with a standard diet without lactoferrin also showed signs of hair loss, decreased vitality, lethargy, tachypnea and anorexia. No abnormal signs were found in mice in both the H5N1-Lac^−^ and H5N1^−^Lac^+^ groups.

### 3.2. Effect of Lactoferrin on the Histopathology of Mice Infected with the H5N1 Influenza Virus

The lactoferrin treatment resulted in a significant improvement in the histopathology of both the lungs and colon. In the H5N1^+^Lac^+^ group, the alveolar septum was slightly widened and inflammatory cell infiltration was mild (Figure 1C), which reversed lung tissue damage caused by influenza to a certain extent. However, for the H5N1^+^Lac^−^ group, the lung tissue structure was destroyed, the alveolar septum was significantly thickened and red blood cells and cellulose were exudated in the alveolar cavity, accompanied by many inflammatory cell infiltrations. The pulmonary histopathological observation of mice showed that structures at all levels in the trachea in the H5N1^−^Lac^−^ and H5N1^−^Lac^+^ groups were intact, no obvious abnormality of the alveolar septum was observed, the capillary was slightly dilated and congested and no other obvious histopathological changes were observed. We also assessed colon histopathology. In both the H5N1^−^Lac^+^ and H5N1^−^Lac^−^ groups, the glandular structure of the colon was neat and had no apparent sign. In contrast, we observed that the intestinal mucosa in infected mice showed layer vascular congestion and mild-to-moderate expansion submucosa edema (Figure 1D).

### 3.3. Lactoferrin Altered the Diversity and Composition of Colon Microbiota

**Figure 2:**
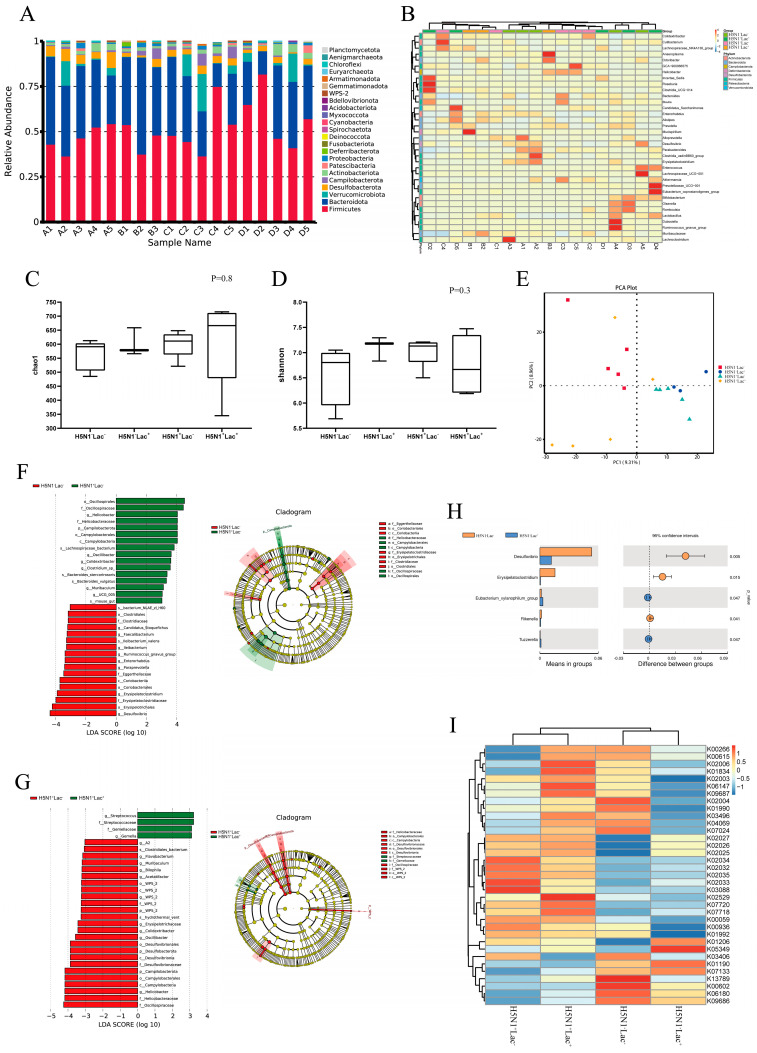
Lactoferrin altered the diversity and composition of colon microbiota in H5N1-infected mice. (**A**) Histogram of relative species abundance at the phylum level. (**B**) Cluster diagram of species abundance at the genus level. (**C**) Chao1 index of colon microbiota. (**D**) Shannon index of colon microbiota. (**E**) PCA plots that visually represent the differences in microbiota composition between groups. Linear discriminant analysis (LDA) effect size scores for taxa that exhibit differential abundance (**F**) between the H5N1^−^Lac^−^ and the H5N1^+^Lac^−^ group; (**G**) between the H5N1^+^Lac^−^ and the H5N1^+^Lac^+^ group. The LDA threshold is 3. (**H**) Differences in microbiota composition between the H5N1^−^Lac^−^ and the H5N1^−^Lac^+^ group by *t*-test. (**I**) Function annotation clustering heat map, which shows the function cluster tree on the left side of the figure. The function annotation information is displayed on the right side of the figure, and the information for each group is shown at the bottom. (Samples A1–A5 belong to the H5N1^−^Lac^−^ group, Samples B1–B3 belong to the H5N1^−^Lac^+^ group, Samples C1–C5 belong to the H5N1^+^Lac^−^ group, and Samples D1–D5 belong to the H5N1^+^Lac^+^ group.).

A total of eighteen mouse samples from four groups were subjected to 16S rRNA amplicons high-throughput sequencing. QIIME2′s class-SkLearn algorithm was used for species annotation of the naive Bayes classifier for each ASV. Twenty-four phyla (twenty-two phyla and two archaea) and forty-one classes were obtained. This also included 99 orders, 170 families, 381 genera, 592 species and 2456 ASVs. According to the analysis of species composition at the phyla level, Firmicutes were the most abundant in each group, with a relative abundance of 36.60–81.99%, followed by *Bacteroidota* (12.89–53.59%), *Verrucomicrobiota* (0.05–20.64%), *Desulfobacterota* (0.74–8.81%) and *Campilobacterota* (0.12–6.59%). As made clear by the histogram of relative abundance, the relative abundance of Firmicutes and Bacteroidota was highest in each group. Compared with the H5N1^−^Lac^−^ group, the relative abundance of Firmicutes and Bacteroidota in the H5N1^+^Lac^−^ group increased and decreased, respectively, but showed no significant difference. Feeding lactoferrin increased the abundance of Firmicutes in the H5N1^+^Lac^+^ group but did not affect Bacteroidota compared with the H5N1^+^Lac^−^ group. The abundance of Firmicutes and Bacteroidota in the H5N1^−^Lac^+^ group did not significantly change after feeding antimicrobial peptides (Figure 2A,B). 

As shown in Figure 2C,D, the alpha diversity, as indicated by the CHAO1 and Shannon indexes, was not significantly changed among all the groups. Principal component analysis (PCA) of the samples from four groups revealed that each group was significantly separated (Figure 2E). These findings suggested that influenza infection had a significant impact on the β diversity of intestinal microflora in mice, and lactoferrin supplementation effectively modulated the microbial community in both infected and uninfected groups. LEfSe analysis of mouse samples showed that *Oscillospiraceae*, *Helicobacter*, *Campilobacterota*, *Desulfovibrio* and *Erysipelatoclostridium* significantly contributed to the differentiation between the H5N1^+^Lac^−^ group and the H5N1^−^Lac^−^ group (Figure 2F). *Streptococcaceae*, *Gemellaceae* and *Desulfovibrionaceae* were identified as the primary biomarkers exhibiting significant statistical differences between the H5N1^+^Lac^−^ group and the H5N1^+^Lac^+^ group (Figure 2G). Feeding lactoferrin resulted in a significant decrease in *Desulfovibrio*, *Erysipelatoclostridium* and *Rikenella* and a significant increase in *Eubacterium_xylanophilum_group* and *Tuzzerella* in the H5N1^−^Lac^+^ group (Figure 2H). PICRUSt2 was used to predict the genomic function of each group, and the KEGG database was used to compare the sequencing results and analyze metabolic pathways. As seen from the clustering heat map, the top 35 functional pathways in abundance had different clustering distributions among each group (Figure 2I).

### 3.4. The Protective Effect of Lactoferrin on the Intestinal Barrier Structure

**Figure 3:**
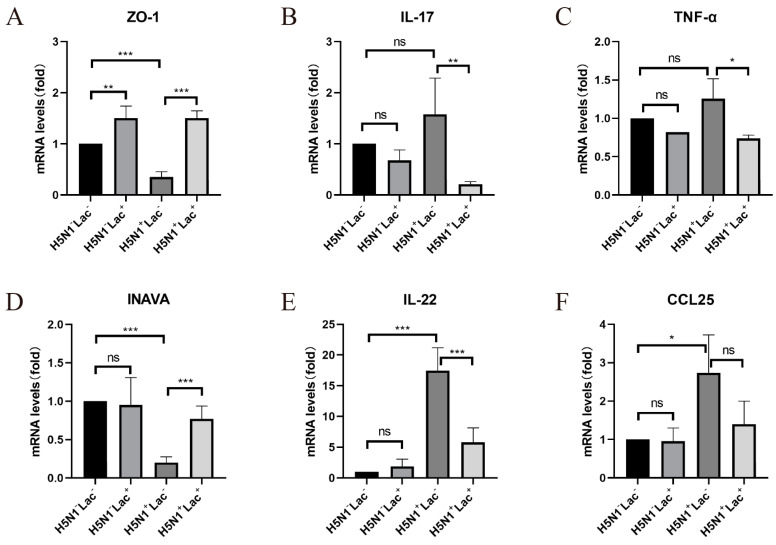
The effect of lactoferrin on the intestinal barrier structure and inflammatory cytokines. The mRNA relative expression of (**A**) ZO-1 in the colon, (**B**) IL-17 in the colon, (**C**) TNF-α in the lung, (**D**) INAVA in the colon, (**E**) IL-22 in the colon, (**F**) CCL25 in the colon, determined via quantitative RT-PCR. The values are represented as mean ± S.D. (ns, not significant, * *p* < 0.05, ** *p* < 0.01 and *** *p* < 0.001, *n* = 5 for all groups).

Compared to the control group, the mRNA expression of the intestinal tight junction protein ZO-1 largely decreased after influenza infection in the H5N1^+^Lac^−^ group (*p* = 0.0002), and significantly increased following antimicrobial peptide feeding (*p* < 0.0001). Additionally, after receiving lactoferrin, the expression of ZO-1 in the H5N1^−^Lac^+^ group also significantly increased compared with the control group (*p* = 0.0050, Figure 3A). Compared with the H5N1^−^Lac^−^ group, the mRNA expression of INAVA in the H5N1^+^Lac^−^ group was significantly decreased (*p* < 0.0001). After adding lactoferrin, the expression of INAVA in the intestinal tract of mice from the H5N1^+^Lac^+^ group was significantly higher than that in the H5N1^+^Lac^−^ group (*p* = 0.0003). However, adding lactoferrin to the diet had no significant effect on the mRNA expression level of INAVA in the H5N1^−^Lac^+^ group (*p* = 0.9792, Figure 3D). 

### 3.5. Effect of Lactoferrin on Inflammatory Cytokines

In the detection of intestine and lung inflammatory cytokines, the mRNA expression of IL-17 in the colon was upregulated by influenza infection, but not statistically significant (*p* = 0.2922), and was significantly downregulated by lactoferrin in the H5N1^+^Lac^+^ group (*p* = 0.0056). In the H5N1^−^Lac^+^ group, the addition of lactoferrin had no significant effect on IL-17 expression compared with the control group (*p* = 0.8300, Figure 3B). After infection with the influenza virus, the expression of tumor necrosis factor (TNF)-α increased in the lungs of the H5N1^+^Lac^−^ group, but there was no statistical significance compared with the H5N1^−^Lac^−^ group (*p* = 0.2732). Its mRNA expression was significantly reduced (*p* = 0.0166, Figure 3C). After influenza infection, IL-22 expression in the intestinal tract was significantly increased (*p* < 0.0001), and the expression level significantly decreased after feeding antimicrobial peptides (*p* = 0.0004), but did not recover to the level of the control group. However, mice in the control group showed no significant change after feeding lactoferrin (*p* = 0.9976, Figure 3E). The mRNA expression of CCL25 in the colon of mice in the H5N1^+^Lac^−^ group was significantly increased compared with the H5N1^−^Lac^−^ group (*p* = 0.0193), and decreased after feeding lactoferrin, but there was no statistical significance (*p* = 0.1065). The addition of lactoferrin in the H5N1^−^Lac^+^ group had no significant effect on CCL25 expression (*p >* 0.9999, Figure 3F).

## 4. Discussion

In this study, the mice infected with the H5N1 virus showed weight loss from day 2, and most of them also showed symptoms such as loss of appetite, decreased vitality, dull hair color, diarrhea and unformed feces. These symptoms were significantly improved after feeding with lactoferrin, and some regained vitality. The H5N1 influenza infection can cause lung tissue damage in a short period of time, which is characterized by capillary dilatation and congestion, significant alveolar thickening and inflammatory cell infiltration [26]. In this study, feeding mice with lactoferrin alleviated vascular dilatation and congestion, reduced inflammatory cell infiltration and improved alveolar septum widening. Only mild dilatation and congestion of blood vessels in the mucosal layer were observed after feeding with lactoferrin. There have been reports indicating that lactoferrin can inhibit SARS-CoV-2 infection in the nanomolar range in cell models [27] and lactoferrin can be a carrier for drug delivery in the treatment of inflammatory bowel disease [28].

The intestinal tract may also experience changes such as villous atrophy, cell necrosis, lymphocyte infiltration and hyperemia after a long enough period of H5N1 infection [29], leading to intestinal ischemia and hypoxia [30]. Hypoxia-inducible factor-1 alpha (HIF-1α) is a protein induced by hypoxia during inflammation and infection, and its upregulation may lead to the loss of tight junction proteins such as ZO-1 [31], thereby damaging the integrity of the intestinal barrier [32]. The results of the q-PCR showed that influenza infection significantly decreased the expression of the intestinal tight junction protein ZO-1, whereas lactoferrin antimicrobial peptide feeding significantly increased its expression. The integrity of the intestinal barrier is maintained by tight junction proteins, including ZO-1, Claudin-1 and Occludin [10]. Among them, ZO-1 is one of the markers of intestinal barrier integrity and has been widely used in the evaluation of intestinal injury in studies conducted on inflammatory effects, especially studying inflammatory bowel disease (IBD). We detected the mRNA expression levels of these three tight junction proteins in the intestinal tracts of mice. Similar to a previous study [33], the expression of ZO-1 in the intestinal tracts of mice was greatly reduced after H5N1 infection, while Claudin-1 did not significantly change. Previous research has shown that lactoferrin B administration significantly improved ZO-1 and Occludin expression and attenuated O157:H7-induced epithelial barrier damage [34]. Lactoferrin intervention improved the expression of Occludin and ZO-1 and repaired the injured intestinal barrier [35]. Antimicrobial peptide feeding upregulated the expression of ZO-1, suggesting that lactoferrin may repair intestinal wall integrity by upregulating ZO-1 protein expression.

Influenza infection induces epithelial cells and macrophages to secrete a variety of pro-inflammatory factors, leading to an influx of inflammatory cells into the infected region [36]. Respiratory tract infection caused by the influenza virus not only causes lung inflammation, but also causes intestinal damage, resulting in an upregulated expression of intestinal pro-inflammatory cytokines [37]. Lung-derived CCR9^+^CD4^+^ memory T cells can migrate to the intestine through the mucosal immune system, where IFN-γ is produced to mediate intestinal inflammation [38], causing intestinal injury with the increased proportion of interleukin-17-producing Th17 cells. A previous study reported that the expression of IL-17 in the intestinal tract increased after influenza infection [21], which is also the finding of our study. INAVA is a gene closely related to intestinal wall integrity. We assessed the expression level of INAVA before and after lactoferrin intake, which serves as an indicator for the degree of intestinal barrier disruption and recovery to a certain extent. The results showed that influenza infection significantly downregulated the expression of INAVA in the intestinal tract of mice, while adding lactoferrin appreciably increased its expression level. In a previous study, INAVA-deficient mice showed intestinal barrier integrity to be defective in a stable state and were more susceptible to mucosal infections [39]. Influenza infection notably downregulated INAVA, and compromised the integrity of the intestinal wall of mice at a time in which intestinal mucosa was more vulnerable to pathogenic bacteria [40]. This finding was in line with our results in the current study, that influenza infection increased the abundance of pathogenic bacteria in the intestinal flora of mice.

In recent years, it has been found that pathogens such as influenza viruses can cause intestinal dysfunction after entering the respiratory system, thus affecting the lung immune response. Therefore, the enteric-lung axis theory has been proposed to describe the close relationship between the two organs [41]. Studies have shown that intestinal flora play a regulatory role in eliciting immune responses against the influenza virus, and it has been proven that the regulation of intestinal flora may be used to treat lung diseases [42]. Similar to other studies using rodents [43,44], our study identified that Firmicutes and Bacteroides have the highest abundance in the intestinal microbiota of mice. After infection with influenza, the abundance of Firmicutes in intestinal microflora increased, but there was no significant difference. Feeding lactoferrin could reduce the abundance of Firmicutes to a certain extent, but had no significant effect on Bacteroides. The increased abundance of Firmicutes and Bacteroides is a manifestation of the disruption of intestinal microecological homeostasis [45]. A higher abundance of Firmicutes had been found in studies of intestinal dysfunction in mice brought on by influenza infection or obesity [13,46]. In this study, influenza infection led to changes in the diversity of intestinal flora in mice, and lactoferrin antimicrobial peptides reversed this change to some extent.

Intestinal microbiota play an important role in the etiology of inflammatory bowel disease [47]. Mucosa-associated bacteria such as *Campylobacter* and *Helicobacter pylori* are thought to play a key role in the initiation and development of Crohn’s disease and ulcerative colitis [48]. Studies have shown that *Campylobacter* has a very diverse pathogenicity, as well as unique genetic and functional characteristics determined by their ability to adhere and invade host cells and secrete toxins, and they have restrictive modification systems associated with virulence [49]. Some studies have reported a positive correlation between gastrointestinal diseases and *H. pylori;* for example, *H. pylori* has been positively correlated with duodenal ulcer, gastric ulcer, gastritis and esophageal cancer [50,51]. *Desulfovibrio*, a sulfate-reducing bacterium, has been reported to be significantly increased in DSS-induced animal enteritis models [52,53]. *Eu. xylanophilus* can produce short-chain fatty acids, which improve host health by lowering pH and inhibiting the growth of harmful intestinal bacteria [54]. In this study, the relative abundance of *Desulfovibrio*, *Erysipelatoclostridium*, *Enterorhabdus* and *Rikenella* was significantly reduced after infection with the H5N1 influenza virus, whereas *Colidextribacter*, *Oscillibacter* and *Tuzzerella* significantly increased at the genus level. In addition, adding lactoferrin to the feed in healthy mice reduced pathogenic bacteria, such as *Desulfovibrio* and *Erysipelatoclostridium*, and increased the abundance of beneficial bacteria, such as *Eu. xylanophilus*.

Compared with the H5N1^−^Lac^−^ group, 133 significant changes were found in the KEGG Orthology (KO) annotation of the H5N1^+^Lac^−^ group. The results showed that the upregulated pathway after influenza infection was more conducive to bacterial colonization and bacterial community information transmission, while antimicrobial peptides promoted the metabolism of bacterial communities and enhanced the absorption and immune function of epithelial cells. Similar to other studies, antimicrobial peptides enhanced the immune function and anti-stress ability of the body [55], and especially improved the disorder of energy metabolism [56]. The main ingredient of the antibacterial peptide used in this study is lactoferrin, which inhibits inflammation, promotes cell metabolism and growth and promotes DNA synthesis [6], which is consistent with our study results. In the case of healthy mice fed with lactoferrin, there were no significant differences in the pathways involved in upregulated and downregulated KO annotation, indicating that the intestinal flora of healthy individuals were in dynamic balance, and antimicrobial peptides did not affect their homeostasis in functional pathways. In summary, this study revealed that lactoferrin could alleviate inflammation and regulate gut microbiota composition in H5N1-infected mice. Further studies are needed to confirm its efficacy in clinical studies.

## 5. Conclusions

**Figure 4:**
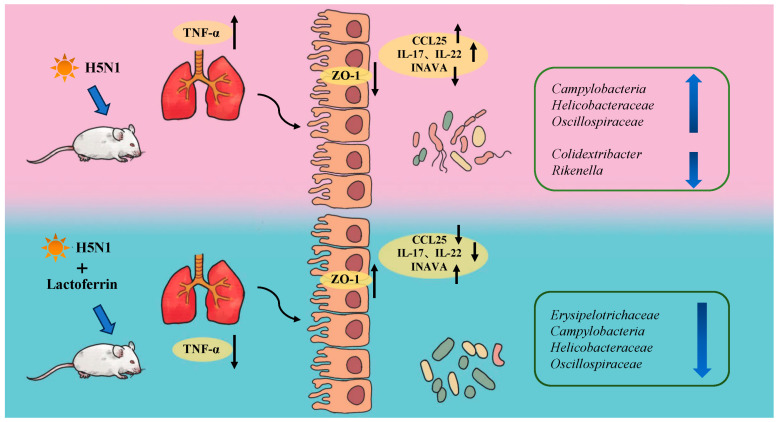
Schematic model summarizing the role of lactoferrin in the regulation of the inflammation and microbiota composition in H5N1-infected mice.

Our findings provided evidence that lactoferrin has anti-inflammatory effects and regulates the composition of gut microbiota in H5N1 influenza virus mice. Influenza infection led to increased levels of inflammation, changes in intestinal flora homeostasis and damage to the integrity of the intestinal wall. Lactoferrin reduced the abundance of harmful bacteria such as *Campylobacter*, repaired the intestinal barrier by upregulating the expression of ZO-1 and INAVA, downregulated the expression of CCL25 and affected the recruitment of intestinal lymphocytes and their migration to the mucosal immune system, thus reducing the levels of intestinal and lung inflammatory factors. The results suggested that lactoferrin modulated the immune response induced by influenza infection through a complex and extensive synergistic relationship among gut microbiota, inflammatory factors and the intestinal barrier (Figure 4).

## Figures and Tables

**Table 1:** q-PCR primers used in this study ^1^.

Gene	Designated Oligo Nucleotides (5′→3′)	GenBank Accession No.
GAPDH-F	CATCACTGCCACCCAGAAGACTG	NM_001289726.2
GAPDH-R	ATGCCAGTGAGCTTCCCGTTCAG	
INAVA-F	TGCCGAAGTTAAATGAAATACC	NM_001405152.1
INAVA-R	CATGATGAGTTTCTGGGAAGAG	
IL-17-F	CAGACTACCTCAACCGTTCCAC	NM_010552.3
IL-17-R	TCCAGCTTTCCCTCCGCATTGA	
TNF-α-F	GGTGCCTATGTCTCAGCCTCTT	NM_001278601.1
TNF-α-R	GCCATAGAACTGATGAGAGGGAG	
CCL25-F	CCGGCATGCTAGGAATTATCA	NM_009138.3
CCL25-R	GGCACTCCTCACGCTTGTACT	
IL-22-F	GCTTGAGGTGTCCAACTTCCAG	NM_016971.2
IL-22-R	ACTCCTCGGAACAGTTTCTCCC	
ZO-1-F	ACTCCCACTTCCCCAAAAAC	NM_001163574.2
ZO-1-R	CCACAGCTGAAGGACTCACA	

^1^ GAPDH, glyceraldehyde-3-phosphate dehydrogenase; INAVA, innate immunity activator; IL-17, interleukin 17; TNF-α, tumor necrosis factor-α; CCL25, C-C motif chemokine ligand 25; IL-22, interleukin 22; ZO-1, Zonula occludens protein 1.

## Data Availability

The original data presented in the study are included in the article, further inquiries can be directed to the corresponding author.
